# The Hahnemannian Monthly and the I. H. A.

**Published:** 1883-08

**Authors:** C. Pearson

**Affiliations:** Washington


### THE HAHNEMANNIAN MONTHLY AND THE I. H. A.

C. Pearson, M. D., Washington.

In an editorial in the July number of the *Hahnemannian Monthly*, the writer, in speaking of a resolution introduced on the last day of the recent session of the American Institute by Dr. Dake, the object of which was undoubtedly to prevent, if possible, the International Hahnemannian Association from holding its meeting at the same time and place as those of the Institute, the following language is used: "It had been known that an *outside* organization of physicians had held its sessions in an adjoining room, and had *attracted*, or *sought to attract*, members from the Institute's meetings. * * * *Moreover, it had just been publicly declared in the Institute* by one of the members of that outside body that a portion of his fellow members were *ready and anxious to effect a schism in the Institute*, and that if they were prevented from holding meetings *in opposition to those of the rightful body*, there was danger that *all* the members of the International Hahnemannian Association would secede from the Institute."*

There is not one word of truth in all this—a pure fabrication, out and out. Take the language just as it reads: "Moreover, it *had just been* publicly declared in the Institute." Now this implies that the declaration which follows (but which, by the way, was never made) was made prior to the introduction of the Dake resolution, and that it was a kind of incentive for its introduction, and the impression and misrepresentation here sought to be made is an effective illustration of the doctrine of total depravity. The remarks of Dr. Smith, the gentleman to whom reference is here made, were called out by the introduction of the resolution and in its discussion; he used no such language as is imputed to him. *He did not* say that "a portion of his fellow members were ready and anxious to effect a schism in the Institute," or anything that could possibly be construed to mean or imply this. The thought and the language are the spontaneous products of the fertile imagination of the writer, and if he thinks that by perverting the truth in this way he can excite prejudice against the International Hahnemannian Association and thereby benefit the Institute, he will find he is hand-

* Italics are ours.

ling a "boomerang." Dr. Smith did *not* say that if we were prevented (!) (prevented is good) "from holding meetings in *opposition* to those of the *rightful* body, there was danger that *all* the members of the International Hahnemannian Association would secede from the Institute." He said nothing of the kind. He simply saw, as did others, the animus of the resolution, and that it was intended, if possible, to cripple the International Hahnemannian Association, and he warned the Institute that if it adopted any such legislation it might tend to drive away "some of its best members." Whether this had the effect, as the writer suggested, of "scaring" the Institute to table the resolution, or whether it saw the ridiculousness of the proposition, and how utterly impossible it would be if adopted to enforce it, we cannot tell, neither can we repeat from memory the exact words of the resolution, but it was something like the following:

"*Resolved*, That the meetings of no other society, or association, shall be held in a hotel where the Institute meets during the sessions of that body."

To this Dr. Pearson offered the following amendment: "That no member be permitted to meet with a number of his friends in his own room during the sessions of the Institute;" stating at the same time that one proposition was just as reasonable and consistent as the other.

The facts are, anticipating trouble, I had engaged a large room for myself on the first floor of the hotel, and paid for the use of it with my own money, and in this room the sessions of the International Hahnemannian Association were held, and it was none of the Institute's business, neither is it the business of the editor of the *Hahnemannian Monthly*, who were in that room, when, how often, or how long they were there, or what business was transacted. We will do the same thing again just whenever we please. We did not "seek to attract" any one from the Institute; on the contrary, if any outsider came in he usually soon felt that he was in the wrong place and not wanted. It is refreshing to hear this editor talking about the Institute's "framing a law which would secure the object it had in view and defend the rights its own money had paid for." *Its own money!* Whose money? Who owns the Institute, anyway? [Dake & Co.—Ed. H. P.] It appears to me the "Internationals" have some stock in it, though it does not seem to be paying a very big per cent. just now. It is playing the baby to talk about the Institute's money, when it receives more from the Internationals in dollars and cents in one year than it returns in useful information in five. We ask no favors of the Institute. We owe it nothing. We do not expect to even attempt to produce a schism, neither do we propose to be coerced, bulldozed, or misrepresented by it as a body or by its individual members. We would advise it, however, to go slow, to feel its way more carefully in the future—to "make haste slowly;" a little less hasty legislation on the last day of the session, when there are only probably twenty or thirty members present, as was the case when the Dudley resolution was passed at Indianapolis. Fight shy of Egbert Guernsey's proposition to strike the word Homœopathy from its name. It remained for a member of the Internationals to denounce this project as it deserves; though it was really not our funeral; for our own part, we feel disposed, should the Institute contemplate suicide, to furnish it with a rope. If it succeeds in getting clear of its name, which is pretty much all it has left of Homœopathy, one of its members says he will clothe himself in sackcloth and ashes and join the Internationals. Poor fellow! Does he not know it is no easy matter to become a member of that body? That if all the members of the Institute were to make application for membership in the International Hahnemannian Association, nine-tenths of them would be black-balled.

No, no, Doctor! Look after your own corpse; we are not hunting *members;* we want Hahnemannians, and will receive no others if we know it. We wage no offensive warfare, but when attacked we have the power and claim the right to defend ourselves, and do not propose to be misrepresented, falsified, or villified without resenting it.

